# Epidemiologic Characteristics of Multimorbidity and Sociodemographic Factors Associated With Multimorbidity in a Rapidly Aging Asian Country

**DOI:** 10.1001/jamanetworkopen.2019.15245

**Published:** 2019-11-13

**Authors:** Lian Leng Low, Yu Heng Kwan, Michelle Shi Min Ko, Cheng Teng Yeam, Vivian Shu Yi Lee, Wee Boon Tan, Julian Thumboo

**Affiliations:** 1 Department of Family Medicine and Continuing Care, Singapore General Hospital, Singapore; 2 Health Services and Research Evaluation, SingHealth Regional Health System, Singapore; 3 Program in Health Services and Systems Research, Duke-NUS Medical School, Singapore; 4 Duke-NUS Medical School, Singapore; 5 Medicine Academic Clinical Program, Singapore General Hospital, Singapore

## Abstract

**Importance:**

Multimorbidity is a growing health care problem in aging societies and is strongly associated with epidemiologic characteristics and sociodemographic factors. Knowledge of these associations is important for the design of effective preventive and management strategies.

**Objectives:**

To determine the association between multimorbidity and sociodemographic factors (age, socioeconomic status [SES], sex, and race/ethnicity) and the association between mental health diseases and physical diseases, as well as their implications for the types and costs of health care use.

**Design, Setting, and Participants:**

This population-based cross-sectional study used deidentified Singapore Eastern Regional Health System data collected between January 1, 2012, and December 31, 2016. Patients who were alive as of January 1, 2016, and residing in the Regional Health System region in 2016 (N = 1 181 024) were included. Patients who had no year of birth records (n = 573), were born in 2017 (n = 93), or died before January 1, 2016 (n = 47 322), were excluded.

**Main Outcomes and Measures:**

Multimorbidity, age, sex, SES, mental health, race/ethnicity, and health care use.

**Results:**

In the study population of 1 181 024 individuals, the mean (SD) age was 39.6 (22.1) years, 51.2% were women, 70.1% were Chinese, 7.1% were Indian, 13.5% were Malayan, and 9.3% were other races/ethnicities. Multimorbidity, present in 26.2% of the population, was more prevalent in female (26.8%; 95% CI, 26.7%-26.9%) than in male (25.6%; 95% CI, 25.5%-25.7%) patients and among patients with low SES (41.6%) than those with high SES (20.1%). Mental health diseases were significantly more prevalent among individuals with low SES (5.2%; 95% CI, 5.1%-5.2%) than high SES (2.1%; 95% CI, 2.0%-2.1%; *P* < .001). The 3 most prevalent disease combinations were chronic kidney disease and hypertension, chronic kidney disease and lipid disorders, and hypertension and lipid disorders. Although chronic kidney disease, hypertension, lipid disorders, and type 1 and/or type 2 diabetes–related diseases had a low cost per capita, the large number of patients with these conditions caused the overall proportion of the cost incurred by health care use to be more than twice that incurred in other diseases.

**Conclusions and Relevance:**

These findings emphasize the association between multimorbidity and sociodemographic factors such as increasing age, lower SES, female sex, and increasing number of mental disorders. Health care policies need to take sociodemographic factors into account when tackling multimorbidity in a population.

## Key Points

**Question:**

What epidemiologic characteristics and sociodemographic factors are associated with multimorbidity in Singapore?

**Findings:**

In this cross-sectional study of 1 181 024 patients, increasing age, lower socioeconomic status, female sex, and increasing number of mental disorders were significantly associated with increasing multimorbidity.

**Meaning:**

Epidemiologic characteristics and sociodemographic factors must be taken into consideration when developing public health policies, and greater efficacy in managing multimorbidity may be derived from preventive health programs.

## Teaser

This cross-sectional study conducted in Singapore examines the association between multimorbidity and sociodemographic factors and the association between mental health diseases and physical diseases, as well as their implications for the types and costs of health care use.

## Introduction

With the global challenge of an aging population and the increasing prevalence of multiple chronic diseases,^1^ a paradigm shift by governments and health care systems is essential in the management of limited resources and increasing medical expenditures. For people with multimorbidity, commonly defined as the same individual concurrently having 2 or more chronic conditions, the single-disease approach in health care delivery is often inefficient and duplicative.^2^ Therefore, this framework will have to evolve to become broader, more integrated, and with better coordination to accommodate patients’ varied clinical needs. The prevalence patterns of multimorbidity are associated with sociodemographic factors such as age^3^ and sex,^4^ ranging from 50% to 98% of people older than 65 years in different studies.^5,6,7^ Multimorbidity is also significantly associated with decreased functional status,^8^ reduced quality of life,^4^ greater use of health care resources,^2,3^ and high mortality rates.^9^

A product of its brisk economic growth from a developing country to a developed country, Singapore’s stable, affordable, and accessible health care infrastructure has also been associated with a rapidly aging population.^10^ Life expectancy is expected to increase globally,^11^ and alongside greater added years of living comes a greater risk of disease for an individual.^12^ In Singapore, 25% of the resident population will be older than 65 years by 2030.^13^ With 16.3% of the Singapore population having more than 1 chronic condition,^14^ the prevalence of multimorbidity in the elderly population and the associated socioeconomic and disease burden are expected to increase significantly.

Although the association between use of health care and multimorbidity has been investigated,^15^ there is a knowledge gap in examining the association between socioeconomic status (SES) and multimorbidity, to our knowledge.^3,16,17^ Also, most studies focused on older populations or hospital populations,^15,18^ despite some studies reporting that the absolute number of people with multimorbidity was higher among those younger than 65 years.^19^ This warrants a need for more studies to examine the associations and implications of multimorbidity across the entire population, to improve health care services. Improved understanding of the interaction between physical diseases and mental health diseases will be critical for health care policy planning, resource allocation, streamlining services, and moving away from a single-disease approach.^20,21^ Therefore, our study aimed to fill these gaps. Using Singapore as a case study of a rapidly aging Asian country provides a unique opportunity to extrapolate our findings to other countries with similar profiles.

## Methods

### Study Design and Population

Our cross-sectional study was conducted using the deidentified, administrative data from the Ministry of Health in Singapore. The data include a total of 1 229 012 individuals residing in the Singapore Eastern Regional Health System (RHS) region, the largest RHS in Singapore.^22^ It provides integrated care for people residing within the Eastern region of Singapore, with coverage encompassing tertiary hospitals, community hospitals, and large primary care polyclinics.^23^ All patients provided written informed consent to participate in this research. Approval for data retrieval was obtained from the Centralized Institutional Review Board. The SingHealth Centralized Institutional Review Board also granted this study ethics approval. This study followed the Strengthening the Reporting of Observational Studies in Epidemiology (STROBE) reporting guideline.

Patients were included in this study if they were alive as of January 1, 2016, and residing in the Eastern RHS region in 2016 (patients who moved out of the region before January 1, 2016, were not included in the data set). Furthermore, patients without year of birth records, born in 2017, or who died before January 1, 2016, were excluded from the data set.

### Data Collection

The Eastern RHS merges all health care data from 3 of the 6 health care groups in Singapore.^23^ Given that all admissions in these health care groups submit claims data, the data set is comprehensive in capturing data, which include all admissions in public and private health care institutions covered under Medisave (national medical savings scheme),^24^ MediShield Life (basic health insurance plan),^25^ and private primary care visits covered under the Community Health Assistance Scheme (CHAS).^26^

All diagnoses were coded based on the *International Statistical Classification of Diseases and Related Health Problems, Tenth Revision*, a system of medical classification for diseases, symptoms, and procedures published by the World Health Organization.^27^ The data set included demographics, chronic disease status, and health care use between 2012 and 2016.

#### Demographic Characteristics

Patient profiles, sex, age, residential status, race/ethnicity, and SES were analyzed to obtain multimorbidity trends across various subgroups. Race/ethnicity was stratified according to the key racial groups in Singapore (namely, Chinese, Indian, Malayan, and others).^28^ The CHAS status was used to define SES. The CHAS is a government subsidies scheme extended to Singapore citizens from lower-income to middle-income households. Depending on household income level, Singapore citizens are given different amounts of subsidies in a bid to make health care affordable for all. Singapore citizens with an individual monthly household income of 1100 Singapore dollars (SGD) (760 US dollars as of December 31, 2016) and below are eligible for a blue CHAS card; Singapore citizens with an individual monthly household income of 1101 to 1800 SGD (761 to 1244 US dollars as of December 31, 2016) are eligible for an orange CHAS card.^26^ Patients who acquired a blue CHAS status were categorized as having low SES, patients who acquired orange CHAS status were categorized as having middle SES, and patients with no CHAS status were categorized as having high SES.

#### Chronic Disease Status and Multimorbidity

In this study, we define multimorbidity as the presence of 2 or more chronic conditions, from a list of 48 conditions, concurrently in an individual (eTable 1 in the Supplement). Chronic diseases were selected based on 3 established indexes—the Singapore Chronic Disease Management Programme, the Charlson Comorbidity Index, and the Elixhauser Index.^29,30,31,32,33^ Binary indicators were created for all chronic diseases.

The proportion of patients with different numbers of multimorbidities was stratified according to their respective age groups. In addition, we also studied the patterns of physical-mental health comorbidities. For the top 10 most prevalent chronic diseases (chronic kidney disease, hypertension, lipid disorders, type 1 and/or type 2 diabetes, osteoarthritis, asthma, coronary heart disease, renal disease, cancer without metastasis, and angina), the respective distributions with selected comorbidities were studied.

#### Health Care Use

Medical services considered for the purpose of this study include nationwide health care use in 2016, subcategorized into primary care clinic visits, specialist outpatient clinic visits, emergency department visits, and inpatient admissions. In Singapore, primary care services are provided by polyclinics (20% of primary care services) and private general practitioners (80% of primary care services).^34^ The CHAS is used in all polyclinics, and about 1650 (of 1700) general practitioners have signed up for the CHAS scheme since its inception in 2012.^35^ About 1.3 million Singaporeans are eligible for the CHAS, and the government disbursed about 154 million SGD in CHAS subsidies to about 650 000 Singaporean individuals.^36^

### Statistical Analysis

Descriptive analyses included proportions, mean (SD) values, cross-tabulations, and data visualizations. We calculated the prevalence of multimorbidity in an age stratification model, varied by sex, race/ethnicity, and SES. Multimorbidity patterns compared against age and mental health multimorbidities were statistically different across the Chinese and Indian races/ethnicities (but not patients who were Malayan or of other races/ethnicities). Hence, post hoc tests were used to conduct pairwise comparisons for Chinese and Indian patients with multimorbidities. Differences in the prevalence of multimorbidity between different variable groups (eg, the prevalence of multimorbidity in female vs male patients) were measured using the χ^2^ test of independence. We used logistic regression to examine the associations between mental health diseases and age, sex, and SES, while adjusting for the number of physical diseases. We identified the top 10 most prevalent chronic diseases, then assessed their co-occurrence with one another on the distribution of health care visits and costs in 2016.

Data cleaning and preparation were conducted on Python, version 2.7 (Python Software Foundation); statistical analyses were conducted using Stata/SE, version 14.0 (StataCorp). In 1-tailed and 2-tailed tests, *P* < .05 was considered statistically significant.

## Results

### Demographic Characteristics

Of the 1 229 012 patients in the data set, 1 181 024 were alive as of January 1, 2016, and residing in the RHS region in 2016 and were included in the analysis (patients who moved out of the region before January 1, 2016, were not included in the data set). Their mean (SD) age was 39.6 (22.1) years, 51.2% were women, 70.1% were Chinese, 7.1% were Indian, 13.5% were Malayan, and 9.3% were other races/ethnicities. A total of 47 988 patient records were excluded based on the following exclusion criteria: (1) no year of birth records (n = 573), (2) born in 2017 (n = 93), and (3) died before January 1, 2016 (n = 47 322).

A total of 51.2% of the sample were female patients, 26.2% of the population had multimorbidity, and 2.9% had both physical diseases and mental health diseases. Overall, there was a higher prevalence of multimorbidity in female patients (26.8%; 95% CI, 26.7%-26.9%) than in male patients (25.6%; 95% CI, 25.5%-25.7%) (Table 1).

**Table 1.** Demographic Characteristics, Multimorbidity, and Physical and/or Mental Health Diseases in Singapore, 2016

Characteristic	No. (%)	No. of Chronic Diseases, Mean (SD)^a^	Proportion, % (95% CI)	
			With Multimorbidity	With ≥1 Physical and/or Mental Health Disease
Population, No.	1 181 024	1.27 (2.14)	26.2 (26.1-26.3)	2.9 (2.8-2.9)
Female	604 137 (51.2)	1.24 (2.05)	26.8 (26.7-26.9)	3.4 (3.403.5)
Male	576 887 (48.9)	1.30 (2.24)	25.6 (25.5-25.7)	2.3 (2.2-2.3)
Age, y				
≤24	332 982 (28.2)	0.23 (0.54)	3.3 (3.3-3.4)	0.6 (0.6-0.6)
25-44	340 309 (28.8)	0.53 (0.98)	11.0 (10.8-11.1)	2.0 (2.0-2.1)
45-64	341 098 (28.9)	1.68 (2.12)	39.2 (39.0-39.4)	3.3 (3.3-3.4)
65-84	149 118 (12.6)	3.92 (2.96)	76.4 (76.2-76.6)	7.0 (6.8-7.1)
≥85	17 517 (1.5)	5.05 (3.78)	76.8 (76.2-77.4)	19.5 (18.9-20.1)
Race/ethnicity				
Chinese	828 119 (70.1)	1.32 (2.16)	27.7 (27.6-27.8)	3.1 (3.1-3.2)
Indian	84 132 (7.1)	1.42 (2.36)	27.8 (27.5-28.1)	3.4 (3.3-3.5)
Malay	158 981 (13.5)	1.36 (2.22)	27.2 (26.9-27.4)	2.4 (2.3-2.4)
Other	109 792 (9.3)	0.64 (1.49)	12.7 (12.5-12.9)	1.5 (1.4-1.6)
Socioeconomic status				
Low	269 959 (22.9)	2.06 (2.68)	41.6 (41.4-41.7)	5.2 (5.1-5.2)
Middle	133 846 (11.3)	1.45 (2.20)	30.8 (30.6-31.1)	3.0 (2.9-3.1)
High	777 219 (65.8)	0.97 (1.83)	20.1 (20.0-20.2)	2.1 (2.0-2.1)
No. of diseases				
0	667 063 (56.5)	NA	NA	0
1	204 525 (17.3)	NA	NA	0.03 (0.02-0.04)
2	88 774 (7.5)	NA	NA	8.2 (8.0-8.4)
3	68 291 (5.8)	NA	NA	8.4 (8.2-8.7)
4	54 644 (4.6)	NA	NA	8.3 (8.1-8.5)
5	33 652 (2.9)	NA	NA	12.2 (11.9-12.6)
6	23 161 (2.0)	NA	NA	14.5 (14.0-14.9)
7	15 406 (1.3)	NA	NA	16.3 (15.7-16.9)
≥8	25 508 (2.2)	NA	NA	25.0 (24.4-25.5)
Health care use in 2016				
Polyclinic visits	438 071 (37.1)	2.21 (2.57)	46.7 (46.5-46.8)	4.6 (4.56-4.7)
GP or CHAS use	187 217 (15.9)	2.70 (2.81)	55.4 (55.1-55.6)	5.6 (5.5-5.7)
Specialist outpatient clinic visits	358 369 (30.3)	2.58 (2.81)	51.3 (51.2-51.5)	6.6 (6.5-6.6)
Emergency department visits	171 101 (14.5)	2.47 (3.14)	43.0 (42.8-43.3)	7.3 (7.2-7.4)
Inpatient admissions	108 544 (9.2)	3.12 (3.48)	52.1 (51.8-52.4)	9.0 (8.8-9.1)

Abbreviations: CHAS, Community Health Assistance Scheme; GP, general practitioner; NA, not applicable.

^a^ Chronic diseases are categorized into physical and mental health diseases. See eTable 1 in the Supplement for a list of chronic diseases. Comparison of means within each variable shows a significant difference (*P* < .05) in the mean number of chronic diseases (*t* test for sex; 1-way analysis of variance for age groups, races/ethnicities, and socioeconomic status).

### Multimorbidity, Age, and SES

**Figure 1.**
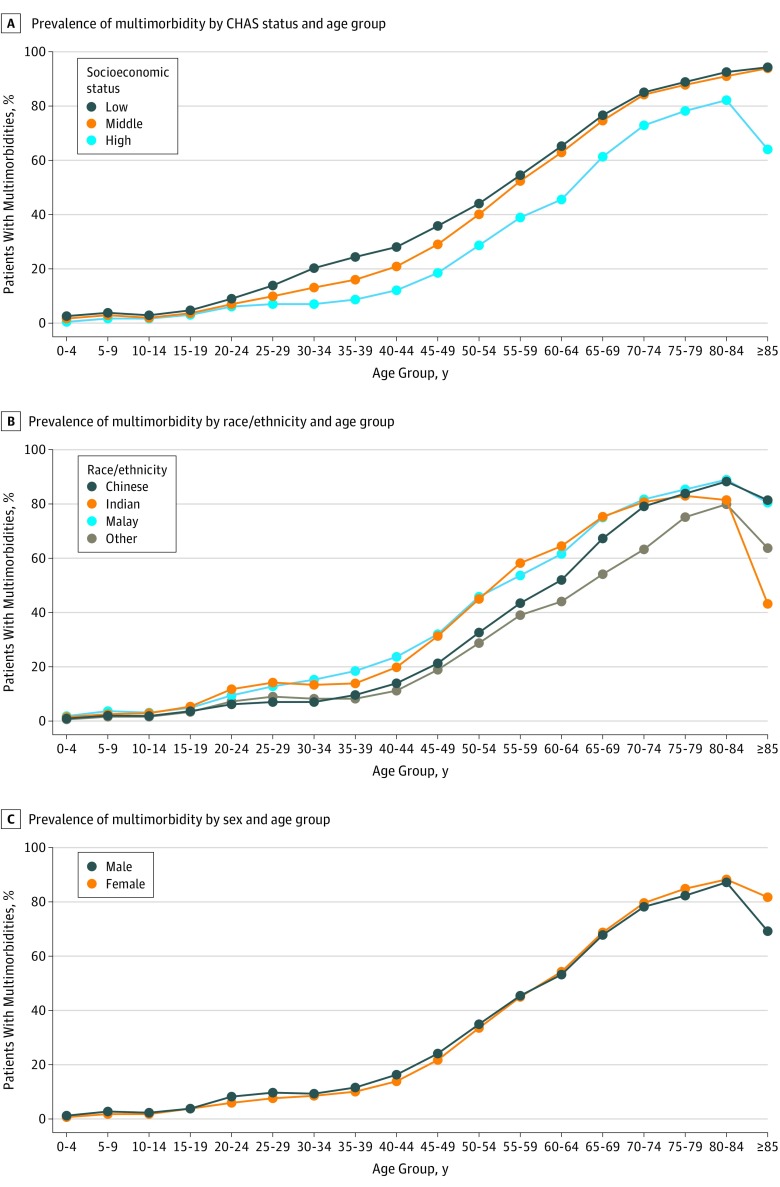
Prevalence of Multimorbidity by Age, Varied by Socioeconomic Status, Race/Ethnicity, and Sex Low socioeconomic status indicates a monthly household income of 1100 Singapore dollars (SGD) and below (for households with income), middle socioeconomic status indicates a monthly household income of 1101 to 1800 SGD (for households with income), and high socioeconomic status indicates a monthly household income of more than 1800 SGD (for households with income). (Exchange Rate as of December 31, 2016, is 1 US dollar = 1.4465 SGD). CHAS indicates Community Health Assistance Scheme.

There was a positive association between mean number of chronic diseases and age. More than 50% of the population had at least 1 chronic disease by the age of 50 years, and more than 50% of the population had multimorbidity by the age of 60 years (eFigure in the Supplement). The proportion of individuals older than 80 years with multimorbidity decreased slightly (Figure 1).

The prevalence of multimorbidity was also associated with SES. Multimorbidity was more prevalent among patients with low SES than patients with high SES; the proportion of multimorbidity among patients with low SES (41.6%) was more than twice that of patients with high SES (20.1%) (Table 1). Adults with low SES between the ages of 40 and 60 years had multimorbidity rates equivalent to their high SES counterparts who were 10 years older (Figure 1A).

### Race/Ethnicity and Age

A χ^2^ test of independence indicated a significant association between race/ethnicity and multimorbidity (χ^2^_3_ = 0.00; n = 1 181 024; *P* < .001). For patients younger than 70 years, there was significantly lower prevalence of multimorbidity among Chinese individuals than Indian individuals (*z* = –17.57; 2-sample *z* test of proportions; *P* < .001). For patients 80 years and older, however, there was a significantly higher prevalence of multimorbidity among Chinese individuals than Indian individuals (*z* = 35.06; 2-sample *z* test of proportions; *P* < .001; Figure 1B).

When age was further stratified into 5-year age bands, we found that male patients younger than 54 years were significantly more likely than female patients to have multimorbidity (*z* = 12.24; 2-sample *z* test of proportions; *P* < .001). Among individuals older than 70 years, this trend reverses, with female patients significantly more likely than male patients to have multimorbidity (*z* = 15.06; 2-sample *z* test of proportions; *P* < .001; Figure 1C).

### Multimorbidity and Mental Health

**Figure 2.**
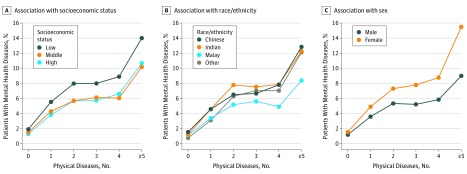
Physical and Mental Health Diseases and the Association With Socioeconomic Status, Race/Ethnicity, and Sex Low socioeconomic status indicates a monthly household income of 1100 Singapore dollars (SGD) and below (for households with income), middle socioeconomic status indicates a monthly household income of 1101 to 1800 SGD (for households with income), and high socioeconomic status indicates a monthly household income of more than 1800 SGD (for households with income). (Exchange Rate as of December 31, 2016, is 1 US dollar = 1.4465 SGD).

Overall, the number of mental health diseases increased with multimorbidity (Figure 2). When stratified by SES, we found that patients with low SES generally had a significantly higher prevalence of mental health diseases than did patients with high SES (5.2% [95% CI, 5.1%-5.2%] vs 2.1% [2.0%-2.1%]; *z* = 288.63; 2-sample *z* test of proportions; *P* < .001; Figure 2A).

Because Singapore represents a racially heterogeneous population, we also examined the association between multimorbidity and health compared across the different races/ethnicities. The prevalence of physical and mental health diseases was found to be different when stratified by race/ethnicity within the population. In comparison with Malayan individuals, Chinese (*z* = 78.78; 2-sample *z* test of proportions; *P* < .001) and Indian individuals (*z* = 54.50; 2-sample *z* test of proportions; *P* < .001) had a higher prevalence of mental health diseases (Figure 2B).

Female patients were also generally more likely than male patients to have mental health diseases (*z* = 150.75; 2-sample *z* test of proportions; *P* < .001; Figure 2C). In the presence of physical diseases, the odds of female patients having a mental health diseases increased across all models (ie, in model 1, which describes results from the logistic regression of demographics on the prevalence of mental health diseases, female patients had 39% higher odds [odds ratio, 1.39; 95% CI, 1.36-1.41] than male patients of having a mental health disease; in model 2, which describes results from the logistic regression of demographics and number of physical diseases on mental health diseases, female patients had 42% higher odds [odds ratio, 1.42; 95% CI, 1.40-1.45] than male patients of having a mental health disease) (eTable 2 in the Supplement).

### Multimorbidity Patterns

eTable 3 in the Supplement shows the prevalence of the 10 most common chronic diseases within the study population. Diseases that were most prevalent in the population were chronic kidney disease (31.9%), hypertension (18.5%), lipid disorders (18.3%), diabetes (8.7%), osteoarthritis (5.7%), asthma (5.1%), coronary heart disease (5.0%), renal disease (3.1%), cancer (without metastasis) (3.1%), and angina (1.9%). The 3 most prevalent disease combinations were chronic kidney disease and hypertension, chronic kidney disease and lipid disorders, and hypertension and lipid disorders.

### Multimorbidity and Health Care Use Patterns

Table 2 shows the distribution of health care use costs per capita incurred from patients with different disease combinations. Although chronic kidney disease, hypertension, lipid disorders, and diabetes-related diseases had a low cost per capita, a large number of patients with these diseases were associated with the overall proportion of health care use costs incurred being more than 2 times of the other diseases.

**Table 2.** Distribution of Health Care Use and Costs in 2016 Across Patients With the Top 10 Most Common Chronic Diseases^a^

Disease	CKD	Hypertension	Lipid Disorder	Diabetes^b^	Osteoarthritis	Asthma	CHD	Renal Disease	Cancer^c^	Angina
**Distribution of No. of Patients, Millions**										
CKD	2.86	NA	NA	NA	NA	NA	NA	NA	NA	NA
Hypertension	1.83	2.10	NA	NA	NA	NA	NA	NA	NA	NA
Lipid disorders	1.84	1.73	2.10	NA	NA	NA	NA	NA	NA	NA
Diabetes^b^	1.00	0.96	1.00	1.09	NA	NA	NA	NA	NA	NA
Osteoarthritis	0.62	0.57	0.55	0.28	0.73	NA	NA	NA	NA	NA
Asthma	0.30	0.22	0.21	0.12	0.10	0.42	NA	NA	NA	NA
CHD	0.59	0.56	0.55	0.33	0.18	0.08	0.62	NA	NA	NA
Renal Disease	0.41	0.39	0.37	0.26	0.12	0.05	0.18	0.43	NA	NA
Cancer^c^	0.36	0.26	0.25	0.14	0.08	0.03	0.08	0.07	0.40	NA
Angina	0.24	0.23	0.23	0.14	0.08	0.04	0.25	0.07	0.03	0.25
**Distribution of Health Care Use Costs, Millions, $**										
CKD	1413	NA	NA	NA	NA	NA	NA	NA	NA	NA
Hypertension	879	960	NA	NA	NA	NA	NA	NA	NA	NA
Lipid disorders	822	770	892	NA	NA	NA	NA	NA	NA	NA
Diabetes^b^	515	500	492	553	NA	NA	NA	NA	NA	NA
Osteoarthritis	285	254	237	138	321	NA	NA	NA	NA	NA
Asthma	141	99	92	59	43	168	NA	NA	NA	NA
CHD	423	405	386	261	114	52	464	NA	NA	NA
Renal Disease	310	298	268	215	85	35	181	324	NA	NA
Cancer^c^	298	202	176	109	56	25	81	65	343	NA
Angina	155	148	144	98	45	20	164	65	27	164
**Distribution of Health Care Use Costs (per capita), $**										
CKD	3753	NA	NA	NA	NA	NA	NA	NA	NA	NA
Hypertension	5073	4390	NA	NA	NA	NA	NA	NA	NA	NA
Lipid disorders	4712	4730	4119	NA	NA	NA	NA	NA	NA	NA
Diabetes^b^	5888	5897	5559	5381	NA	NA	NA	NA	NA	NA
Osteoarthritis	5838	5893	5636	7105	4791	NA	NA	NA	NA	NA
Asthma	4792	5778	5688	7055	6490	2776	NA	NA	NA	NA
CHD	8413	8573	8113	10 071	9742	9981	7916	NA	NA	NA
Renal Disease	9630	10 185	9811	11 648	10 759	11 200	15 194	8868	NA	NA
Cancer^c^	10 986	10 942	10 183	12 193	10 370	11 364	14 749	16 188	9527	NA
Angina	7672	8207	7746	9764	9458	9085	7252	15 145	13 940	7252

Abbreviations: CHD, coronary heart diseases; CKD, chronic kidney diseases; NA, not applicable.

^a^ Exchange rate as of December 31, 2016, is 1 US dollar = 1.4465 Singapore dollars.

^b^ Type 1 and/or type 2 diabetes.

^c^ Cancer without metastasis.

We assessed the types of health care use costs per capita incurred from patients with different chronic disease diagnosis (top 10 most prevalent chronic diseases) in Table 3. The highest polyclinic costs per capita were incurred among patients with diabetes and the highest comorbid costs were incurred among patients with diabetes and renal disease comorbidities. The highest general practitioner visit costs (per capita) were incurred for osteoarthritis-related chronic diseases, and the highest comorbid costs were incurred among patients with osteoarthritis and asthma comorbidities. The highest specialist outpatient clinic visit costs were incurred among patients with cancer, and the highest comorbid costs were incurred among patients with cancer and chronic kidney disease comorbidities. The highest emergency department costs were incurred for angina-related chronic diseases, and the highest comorbid costs were incurred among patients with angina and renal disease comorbidities. The highest inpatient admission costs were incurred among patients with coronary heart disease and renal disease, and the highest comorbid costs were incurred among patients with cancer and renal disease comorbidities.

**Table 3.** Distribution of Different Health Care Use Costs (per Capita) in 2016, Incurred by Patients With the Top 10 Most Common Chronic Diseases^a^

Disease	CKD	Hypertension	Lipid Disorder	Diabetes^b^	Osteoarthritis	Asthma	CHD	Renal Disease	Cancer^c^	Angina
**Distribution of Costs From Polyclinic Visits**										
CKD	290.16	NA	NA	NA	NA	NA	NA	NA	NA	NA
Hypertension	494.63	407.23	NA	NA	NA	NA	NA	NA	NA	NA
Lipid disorders	508.27	495.69	431.42	NA	NA	NA	NA	NA	NA	NA
Diabetes^b^	652.64	631.88	639.89	574.08	NA	NA	NA	NA	NA	NA
Osteoarthritis	488.32	501.31	523.08	673.53	389.73	NA	NA	NA	NA	NA
Asthma	361.89	492.90	529.81	654.26	494.83	212.91	NA	NA	NA	NA
CHD	485.16	498.21	502.98	614.20	559.10	562.41	425.65	NA	NA	NA
Renal Disease	535.46	567.83	603.87	694.71	599.83	582.91	540.30	483.67	NA	NA
Cancer^c^	317.95	409.09	440.05	546.26	420.60	407.25	427.87	457.87	250.35	NA
Angina	475.06	510.28	504.46	630.04	563.75	557.01	433.98	564.18	446.58	433.98
**Distribution of Costs From General Practitioner Visits**										
CKD	66.26	NA	NA	NA	NA	NA	NA	NA	NA	NA
Hypertension	113.41	149.65	NA	NA	NA	NA	NA	NA	NA	NA
Lipid disorders	106.31	160.91	136.92	NA	NA	NA	NA	NA	NA	NA
Diabetes^b^	111.99	159.76	146.39	143.86	NA	NA	NA	NA	NA	NA
Osteoarthritis	177.04	244.87	228.16	234.78	187.39	NA	NA	NA	NA	NA
Asthma	137.56	268.57	252.45	240.91	321.54	115.99	NA	NA	NA	NA
CHD	107.73	126.45	121.90	132.02	204.36	214.47	109.04	NA	NA	NA
Renal Disease	121.93	142.36	142.58	139.84	221.56	219.35	131.97	122.92	NA	NA
Cancer^c^	99.07	158.01	151.98	157.57	210.96	208.09	133.95	137.34	98.18	NA
Angina	102.46	119.41	114.15	124.02	184.70	188.06	102.46	128.82	138.11	102.46
**Distribution of Costs From Specialist Outpatient Clinic Visits**										
CKD	868.82	NA	NA	NA	NA	NA	NA	NA	NA	NA
Hypertension	1053.53	894.80	NA	NA	NA	NA	NA	NA	NA	NA
Lipid disorders	1032.96	963.05	898.88	NA	NA	NA	NA	NA	NA	NA
Diabetes^b^	1131.74	1073.72	1048.63	1016.23	NA	NA	NA	NA	NA	NA
Osteoarthritis	1221.78	1142.16	1146.59	1264.46	996.83	NA	NA	NA	NA	NA
Asthma	1010.74	1114.55	1160.12	1263.23	1245.83	604.97	NA	NA	NA	NA
CHD	1368.33	1330.67	1312.99	1450.86	1558.63	1556.49	1221.75	NA	NA	NA
Renal Disease	1477.70	1485.80	1462.88	1599.75	1552.66	1617.09	1797.61	1352.80	NA	NA
Cancer^c^	2803.65	2494.36	2497.33	2607.80	2410.32	2557.68	2631.57	2739.32	2275.36	NA
Angina	1438.79	1465.55	1433.11	1583.84	1736.61	1670.58	1326.62	1986.24	3060.17	1326.62
**Distribution of Costs From Emergency Department Visits**										
CKD	159.91	NA	NA	NA	NA	NA	NA	NA	NA	NA
Hypertension	177.71	149.73	NA	NA	NA	NA	NA	NA	NA	NA
Lipid disorders	168.37	163.45	143.52	NA	NA	NA	NA	NA	NA	NA
Diabetes^b^	206.19	199.79	191.93	183.26	NA	NA	NA	NA	NA	NA
Osteoarthritis	222.44	216.17	209.02	268.92	174.58	NA	NA	NA	NA	NA
Asthma	281.23	257.07	262.02	326.68	307.82	167.23	NA	NA	NA	NA
CHD	310.32	304.49	293.56	353.45	403.52	476.95	275.83	NA	NA	NA
Renal Disease	329.30	337.51	336.61	385.87	418.26	520.35	526.95	298.54	NA	NA
Cancer^c^	218.28	245.58	236.18	283.59	275.52	293.94	401.61	421.44	173.53	NA
Angina	354.19	361.56	346.70	422.18	485.33	584.05	324.77	623.71	474.80	324.77
**Distribution of Costs From Inpatient Admissions**										
CKD	2368.12	NA	NA	NA	NA	NA	NA	NA	NA	NA
Hypertension	3233.99	2788.95	NA	NA	NA	NA	NA	NA	NA	NA
Lipid disorders	2896.33	2947.17	2508.50	NA	NA	NA	NA	NA	NA	NA
Diabetes^b^	3785.91	3832.23	3532.45	3463.51	NA	NA	NA	NA	NA	NA
Osteoarthritis	3728.02	3788.90	3528.93	4663.34	3042.22	NA	NA	NA	NA	NA
Asthma	3000.72	3644.95	3483.50	4569.62	4119.89	1675.01	NA	NA	NA	NA
CHD	6141.38	6313.11	5881.41	7520.57	7016.66	7170.35	5884.04	NA	NA	NA
Renal Disease	7165.94	7651.98	7265.52	8828.12	7966.66	8259.84	12 197.16	6610.22	NA	NA
Cancer^c^	7547.31	7635.00	6857.87	8597.60	7052.20	7897.49	11 154.04	12 432.44	6729.61	NA
Angina	5301.25	5749.84	5347.60	7004.39	6487.97	6085.67	5064.38	11 841.65	9820.56	5064.38

Abbreviations: CHD, coronary heart diseases; CKD, chronic kidney diseases; NA, not applicable.

^a^ Exchange rate as of December 31, 2016, is 1 US dollar = 1.4465 Singapore dollars.

^b^ Type 1 and/or type 2 diabetes.

^c^ Cancer without metastasis.

## Discussion

The Singapore Eastern RHS is Singapore’s largest RHS, providing integrated care in tertiary hospitals, community hospitals, and large primary care polyclinics. Our analysis of the Singapore Eastern RHS data set provided evidence that the prevalence of multimorbidity is significantly associated with age, female sex, low SES, and race/ethnicity. Such an understanding of the epidemiologic characteristics and the implications of multimorbidity is necessary for better risk stratification of multimorbidity, integrated coordination of multiple appointments for patients, and more effective communication among health care professionals to better manage patients’ varied clinical needs.

Our study found that multimorbidity in Singapore is more prevalent than in many countries worldwide,^37^ with more than one-fourth of the population having multimorbidity. This proportion increases more than 50% by the age of 60 years. The increase in the number of chronic conditions with age is not surprising, and similar trends have also been found in countries such as Ireland^38^ and Scotland.^19^ In the oldest age group (>80 years), the proportion of individuals with multimorbidity decreased slightly. Increasing multimorbidity is associated with shorter life expectancy,^39^ and the decrease in the number of individuals with multimorbidity in the older age groups is associated with a dampening of the prevalence of multimorbidity within the cohort. However, when we further stratified the association between age and multimorbidity by sex, a trend was seen: male patients younger than 54 years were significantly more likely than female patients to have multimorbidity, but this trend reversed among individuals older than 70 years. This finding supports the age-sex paradox found across many societies,^40^ in which older men have fewer multimorbidities than their counterparts yet have a higher mortality rate. With the onset of multimorbidity occurring at an earlier age in male patients than in female patients, early intervention and management of multimorbidity should also be considered for the male subpopulation. More studies may also be conducted to investigate the protective factors associated with delayed multimorbidity patterns among female patients. Also, future studies may investigate the prevalence and different combinations of specific chronic conditions in patients with multimorbidity in different age groups and SES in Singapore to reduce the health care burden.

Consistent with the trends found in other countries,^41,42^ our study found that multimorbidity and SES share a statistically significant negative association, with the proportion of patients with multimorbidity in the lowest socioeconomic group double that of patients in the highest socioeconomic group. The results from this study are congruent with an earlier study that showed a higher prevalence of multimorbidities among the lower socioeconomic class in Singapore.^43^ It would be worthwhile to evaluate the effectiveness of health and social schemes for the socioeconomically deprived, as well as how resources and services can be streamlined to better manage multimorbidity in this subpopulation.

Our results revealed that Malayan individuals with physical comorbidities have a significantly lower prevalence of mental health diseases than Chinese and Indian individuals with physical comorbidities. Previous studies have shown that Malayan ethnicity is associated with a lower risk of multimorbidity compared with Chinese ethnicity,^14,44^ and Indian individuals have a higher risk of mental-physical multimorbidity.^44^ One possible explanation might be that Malayan individuals with physical comorbidities rely on informal support networks and more helpful coping strategies^45^ and therefore have a significantly lower prevalence of mental health diseases. Taken together, our results suggest that ethnicity may play a role in the risk of having physical and mental multimorbidity, and future studies should be performed to elucidate the reasons for this.

A sociodemographic gradient can also be extended to the prevalence of mental health diseases among patients with multimorbidities. It is well established that mental health diseases occur more frequently among patients with a greater number of physical conditions.^46,47^ Our study found that patients with low SES had significantly more frequent mental health diseases compared with patients with high SES. Such demographic information is useful for health care policy planning and service customization, which may include directing resources to address mental health diseases among patients with low SES, and may even be extended to countries with similar aging profiles^48^ (eg, Scotland,^19^ Australia,^49^ and Hong Kong^50^).

### Limitations

One limitation of using a population database is the heavy reliance of the results on the quality of data recording. Recognizing this potential shortfall, we chose to use a comprehensive database of patients registered with Singapore Eastern RHS in Singapore. All chronic diseases were coded according to *International Statistical Classification of Diseases and Related Health Problems, Tenth Revision* guidelines,^51^ which has been shown to be a robust system capable of supporting detailed reporting in clinical settings. Furthermore, we selected a comprehensive list of 48 conditions to improve the representativeness of the results from this study, based on 3 established indexes—the Singapore Chronic Disease Management Programme, the Charlson Comorbidity Index, and the Elixhauser Index. Given that a small fraction of general practitioners have not signed up for the CHAS (50 of 1700), another limitation of our study may be that our data set is not comprehensive enough to cover all health care services.

Another limitation is that our study used binary indicators to tabulate the number of chronic conditions for each patient in our study population. As such, all conditions in our list were given equal weight and importance. In reality, however, the type, severity, and combination of chronic diseases vary the clinical prognosis for individual patients. In an attempt to mitigate this, we were careful to analyze health care use patterns based on specific combinations of morbidities. Among patients with 2 morbidities, for example, the cost varies drastically depending on the combination of morbidities. Similarly, the types of health care services used by patients with different multimorbidity combinations are also very different, as evident from the health care use patterns in the study sample.

## Conclusions

This study found associations between multimorbidity and factors such as age, sex, race/ethnicity, and socioeconomic differences. The prevalence of multimorbidity increases with age, lower SES, and female sex. We also found a positive association between physical and mental comorbidities. Through identification of the most common chronic diseases and their associated costs, we were able to show the health care service use patterns of patients with different multimorbidities. These findings suggest that holistic management of multimorbidity is warranted, and care must be customized to meet the needs of patients with different multimorbidity patterns.

## Notes

Supplement. **eTable 1.** List of Chronic Diseases, Physical, and Mental Comorbidities **eTable 2.** Logistic Regression of Demographics and Number of Physical Diseases on the Prevalence of Mental Health Diseases **eTable 3.** Distribution of Patients Diagnosed With the Top 10 Most Common Chronic Diseases **eFigure.** Proportion (%) of Patients by Age Groups With Chronic Disease Disorders
