# Error in Methods

**DOI:** 10.1001/jamanetworkopen.2021.1476

**Published:** 2021-02-16

In the Original Investigation titled “Epidemiologic Characteristics of Multimorbidity and Sociodemographic Factors Associated With Multimorbidity in a Rapidly Aging Asian Country,”^1^ published November 13, 2019, there was an error in the first paragraph of Methods. The fourth sentence of the first paragraph should have read that the need for informed consent was waived as the data were fully deidentified. This article has been corrected.^1^
