# Supplementary material for: Epidemiologic Characteristics of Multimorbidity and Sociodemographic Factors Associated With Multimorbidity in a Rapidly Aging Asian Country
Source: JAMA Netw Open. 2019 Nov 13;2(11):e1915245. doi: 10.1001/jamanetworkopen.2019.15245 (PMC6902794; doi:10.1001/jamanetworkopen.2019.15245)
Supplement: Supplement. — eTable 1. List of Chronic Diseases, Physical, and Mental Comorbidities eTable 2. Logistic Regression of Demographics and Number of Physical Diseases on the Prevalence of Mental Health Diseases eTable 3. Distribution of Patients Diagnosed With the Top 10 Most Common Chronic Diseases eFigure. Proportion (%) of Patients by Age Groups With Chronic Disease Disorders [file jamanetwopen-2-e1915245-s001.pdf]

Supplementary Online Content

Low LL, Kwan YH, Ko MSM, et al. Epidemiologic characteristics of multimorbidity and sociodemographic factors associated with multimorbidity in a rapidly aging Asian country. *JAMA Netw Open.* 2019;2(11):e1915245. doi:10.1001/jamanetworkopen.2019.15245

eTable 1. List of Chronic Diseases, Physical, and Mental Comorbidities
eTable 2. Logistic Regression of Demographics and Number of Physical Diseases on the Prevalence of Mental Health Diseases
eTable 3. Distribution of Patients Diagnosed With the Top 10 Most Common Chronic Diseases
eFigure. Proportion (%) of Patients by Age Groups With Chronic Disease Disorders

This supplementary material has been provided by the authors to give readers additional information about their work.

© 2019 Low LL et al. *JAMA Network Open.*

eTable 1. List of Chronic Diseases, Physical, and Mental Comorbidities

**Diseases**	**Chronic Diseases**	**Physical comorbidity**	**Mental comorbidity**
Angina	1	1	
Anxiety	1		1
Arrhythmia	1	1	
Asthma	1	1	
Atrial Fibrillation	1	1	
Benign Prostatic Hyperplasia(BPH)	1	1	
Chronic Obstructive Pulmonary Disease	1	1	
Coronary Heart Disease	1	1	
Diabetes Mellitus	1	1	
Lipid disorders	1	1	
Epilepsy	1	1	
Haemorrhagic Stroke	1	1	
Heart Failure	1	1	
Hip Fracture	1	1	
Hypertension	1	1	
Hyperthyroidism	1	1	
Hypothyroidism	1	1	
Ischemic Stroke	1	1	
Myocardial infarction	1	1	
Nephritis	1	1	
Nephrosis	1	1	
Osteoarthritis	1	1	
Osteoporosis	1	1	
Parkinson's Disease	1	1	
Peripheral Vascular Disease	1	1	
Psoriasis	1	1	
Renal Disease	1	1	
Respiratory Failure	1	1	
Rheumatoid Arthritis	1	1	
Secondary Hypertension	1	1	
Spine Fracture	1	1	
Stroke	1	1	
Bipolar disorder	1		1
Dementia	1		1
General Anxiety Disease	1		1
Major Depression	1		1
Schizophrenia	1		1
Cancer (w/o metastasis)	1	1	
Metastatic Carcinoma	1	1	
Moderate Liver Disease	1	1	
Severe Liver Disease	1	1	
Coronary Artery Bypass Graft	1	1	
Percutaneous Coronary Intervention	1	1	
CKD on Dialysis or pre-Dialysis	1	1	
Kidney Transplant	1	1	
Major Lower Extremity Amputation	1	1	
Minor Lower Extremity Amputation	1	1	
Chronic Kidney Disease	1	1	

”1” Indicates the diseases were categorized under “Chronic diseases”, “Physical comorbidity” and/or “Mental comorbidity”.

© 2019 Low LL et al. *JAMA Network Open.*

eTable 2. Logistic Regression of Demographics and Number of Physical Diseases on the Prevalence of Mental Health Diseases

	Any mental health diseases (unadjusted OR, 95% CI)			Model 1: Any mental health diseases (*adjusted OR, 95% CI)			Model 2: Any mental health diseases (*adjusted OR, 95% CI)		
**Age**	1.03	[1.03,1.03]	p < 0.001	1.03	[1.03,1.03]	p < 0.001	1.01	[1.01,1.01]	p < 0.001
**Female**	1.47	[1.44,1.50]	p < 0.001	1.39	[1.36,1.41]	p < 0.001	1.42	[1.40,1.45]	p < 0.001
**Male**	-			-			-		
**SocioEconomic status**									
Low	2.16	[2.11,2.20]	p < 0.001	1.71	[1.68,1.75]	p < 0.001	1.41	[1.38,1.44]	p < 0.001
Middle	1.32	[1.28,1.36]	p < 0.001	1.25	[1.21,1.29]	p < 0.001	1.08	[1.04,1.11]	p < 0.001
High	-			-			-		
**Race group**									
Indian	1.01	[0.98,1.05]	p = 0.53	1.17	[1.13,1.22]	p < 0.001	1.06	[1.02,1.10]	p = 0.003
Malay	0.73	[0.71,0.75]	p < 0.001	0.83	[0.81,0.86]	p < 0.001	0.74	[0.72,0.76]	p < 0.001
Others	0.50	[0.47,0.52]	p < 0.001	0.75	[0.72,0.79]	p < 0.001	0.78	[0.74,0.81]	p < 0.001
Chinese	-			-			-		
**Number of physical disorders**									
0	-			-			-		
1	3.18	[3.08,3.27]	p < 0.001	-			2.83	[2.74,2.91]	p < 0.001
2	4.84	[4.68,5.01]	p < 0.001	-			3.52	[3.40,3.65]	p < 0.001
3	5.00	[4.82,5.19]	p < 0.001	-			3.21	[3.08,3.34]	p < 0.001
4	5.67	[5.45,5.89]	p < 0.001	-			3.42	[3.28,3.57]	p < 0.001
≥ 5	9.76	[9.49,10.04]	p < 0.001	-			5.55	[5.35,5.75]	p < 0.001

* Comparison of odds ratio within each variable shows a significant difference (p<0.05) in the prevalence of mental health diseases.

Null hypothesis indicates that there is no relationship between predictors and prevalence of mental health diseases. All models were adjusted based on the predictors as follow: Model 1= Demographics; Model 2= Model 1 and number of physical diseases.

© 2019 Low LL et al. *JAMA Network Open.*

eTable 3. Distribution of Patients Diagnosed With the Top 10 Most Common Chronic Diseases

	Chronic Kidney Disease	Hypertension	Lipid disorders	Diabetes Mellitus	Osteo-arthritis	Asthma	Coronary Heart Disease	Renal Disease	Cancer (w/o metastasis)
**Chronic Kidney Disease**	-	-	-	-	-	-	-	-	-
**Hypertension**	173,279.00	-	-	-	-	-	-	-	-
**Lipid disorders**	174,338.00	162,835.00	-	-	-	-	-	-	-
**Diabetes Mellitus**	87,497.00	84,745.00	88,574.00	-	-	-	-	-	-
**Osteoarthritis**	48,831.00	43,055.00	42,018.00	19,470.00	-	-	-	-	-
**Asthma**	29,458.00	17,066.00	16,226.00	8,336.00	6,642.00	-	-	-	-
**Coronary Heart Disease**	50,302.00	47,258.00	47,597.00	25,883.00	11,752.00	5,168.00	-	-	-
**Renal Disease**	32,190.00	29,293.00	27,314.00	18,423.00	7,938.00	3,126.00	11,898.00	-	-
**Cancer (w/o metastasis)**	27,101.00	18,417.00	17,328.00	8,975.00	5,414.00	2,215.00	5,518.00	4,037.00	-
**Angina**	20,245.00	17,994.00	18,623.00	10,024.00	4,751.00	2,255.00	22,554.00	4,296.00	1,931.00

© 2019 Low LL et al. *JAMA Network Open.*

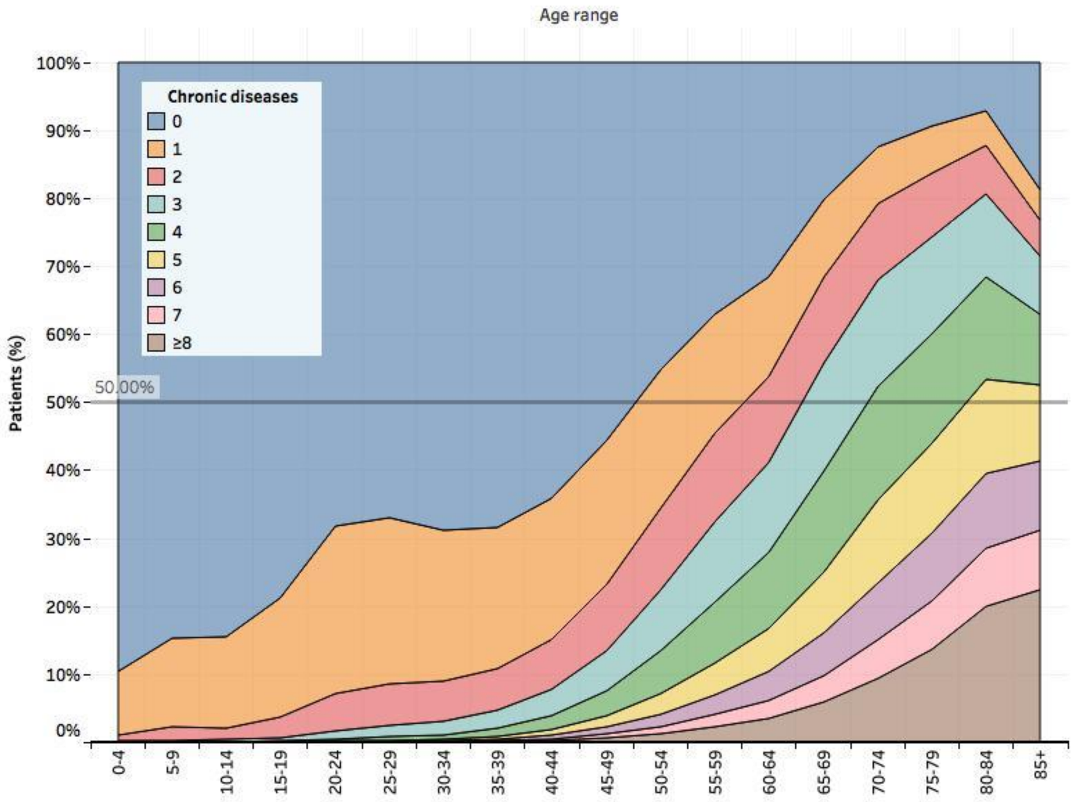

eFigure. Proportion (%) of Patients by Age Groups With Chronic Disease Disorders

© 2019 Low LL et al. *JAMA Network Open.*
